# Lack of association of acute phase response proteins with hormone levels and antidepressant medication in perimenopausal depression

**DOI:** 10.1186/1471-244X-14-164

**Published:** 2014-06-04

**Authors:** Sokratis E Karaoulanis, Katerina A Rizouli, Andreas A Rizoulis, Nikiforos V Angelopoulos

**Affiliations:** 1 Department of Psychiatry, University Hospital of Larissa, University of Thessalia, Mezourlo, Larissa, P.O. Box 41110, Greece; 2 Department of Immunology, University Hospital of Larissa, University of Thessalia, Larissa, Greece; 3 Department of Endocrinology, University Hospital of Larissa, University of Thessalia, Larissa, Greece

**Keywords:** Perimenopause, Depression, Acute-phase response proteins, Reproductive hormones, Selective serotonin reuptake inhibitors

## Abstract

**Background:**

Major depression is associated with higher plasma levels of positive acute-phase proteins, as well as with lower plasma levels of negative acute-phase proteins. The aim of this study is to examine the levels of acute-phase response proteins and whether these levels are influenced by reproductive hormones and antidepressant medication in the perimenopausal depression.

**Methods:**

Sixty-five women (age range: 40–58 years old) participated in this study. All women were in the perimenopausal phase. The diagnosis of depression was made through a psychiatric interview and with the aid of Hamilton Depression Rating Scale 17 (HAM-D 17). The acute-phase response proteins, such as haptoglobin (HP), transferrine (TRf), α1-antitrypsin, complement protein 3 (C3), complement protein 4 (C4) and C-reactive protein (CRP) and the reproductive hormones, for example follicle-stimulating hormone (FSH), luteinizing hormone (LH) and estradiol (E2), were analyzed using standard laboratory methods. Pearson’s correlations were applied to evaluate the relationship between acute-phase proteins and hormones.

**Results:**

Perimenopausal women were divided into three groups. The first group consisted of normal controls, the second one involved depressed perimenopausal women, who were taking selective serotonin reuptake inhibitors (SSRIs), and the third one included depressed women that were not treated with SSRIs. Depressed women in perimenopause, when being compared to non-depressed women, did not differ as to serum levels of acute-phase proteins. There was a positive correlation between HP and E2 in depressed perimenopausal women, who were not taking SSRIs.

**Conclusions:**

The lack of association between acute-phase proteins and depressive mood mentioned in this study does not support previous findings in patients with major depression. This negative finding in perimenopausal depression indicates either the absence or a more complex nature of the interactions between acute-phase proteins, low-grade inflammation and depression. The hormonal profile of women is a part of this complexity, because it seems that in perimenopause the hormonal changes are accompanied by changes of acute-phase response proteins. Particularly, in perimenopausal depression, there is an interaction between HP and E2. Therefore, it seems that perimenopause is a period of a woman’s life during which hormonal, immune and metabolic changes occur and interact with each other making women vulnerable to depression.

## Background

Changes in the immune system of patients suffering from major depression have been reported extensively in the literature. Several studies provided evidence for inflammatory reactions in major depression (MD) [1-3]. Due to its crucial role in acute inflammatory reactions of the body, great attention has been paid to the monocyte-macrophage system.

During immune responses, monocytes (similarly to macrophages) do not just exert local effects. Cytokines, produced by monocytes, exert far-reaching effects on the body. They increase body temperature and stimulate hepatocytes so that acute-phase proteins are produced (e.g. haptoglobin, C-reactive protein, α_1_-macroglobulin). These proteins activate the complement system and opsonize exogenous organisms, such as bacteria.

C-reactive protein (CRP) is one of the most frequently measured acute-phase proteins in clinical medicine. Several studies reported increased serum levels of CRP in patients suffering from MD [4-11], while in other investigations, no differences in CRP serum levels between patients with MD and healthy controls could be found [12-14].

Haptoglobin (HP) is the most frequently studied acute-phase protein in MD, which has provided the most consistent results. Several groups demonstrated increased HP serum levels in MD [8,13-21].

Another acute-phase protein α_2_-macroglobulin (A2M) was found to be significantly higher in patients with acute MD after 6 weeks of clinical treatment compared to healthy controls [8]. Maes et al. [14] reported normal A2M in MD with a trend towards lower levels in melancholia.

Obviously, there is a lack of consistency in the responses of the investigated immune parameters. One reason for this might be that the diagnostic group of MD, classified according to DSM-IV-TR criteria, is simply much heterogeneous. Few researchers have attempted to deal with this problem. Some others have tried to determine associations between immune parameters and severity or type of depression [13,18,22-24], or they have sought and found correlations between psychopathological features and immune function [25]. Another approach has been the study of immunological changes at different stages during the course of the disease [8,26,27].

The DSM-IV-TR classification category of MD covers different kinds of depression but not all of them. The subtypes of depression do not just differ quantitatively; they also differ as far as the quality of the symptoms is concerned [28].

Perimenopausal depression is a type of depression that is not included in the DSM-IV-TR. Throughout most of their lives, women are at greater risk of depression compared to men. The perimenopause and early postmenopausal period have been considered as a “window of vulnerability”, during which physical and emotional discomforts emerge as well as hormonal changes can lead to the appearance of depression. The mechanisms that are responsible for the development of depression in perimenopausal women remain unclear. Studies identified an increased risk of clinical depression, particularly among women with a history of depression [29-32] Although a history of depression continues to be the strongest predictor of a depressive episode, several studies identified depressed mood in perimenopausal women with no history of depression and also identified associations between depression and changes in the hormonal milieu [31-33].

Inflammation levels fluctuate throughout a woman’s life according to hormonal changes that occur due to the phase of menstrual cycle, the use of hormonal contraceptives, the menopause and the use of estrogens [34-36], which might influence the relationship between depression and inflammation.

The first aim of this study is to investigate whether positive acute-phase proteins (haptoglobin, α_1-_antitrypsin and C-reactive protein) and proteins of the complement (C3 and C4) increase, while negative acute-phase proteins, such as transferrin, decrease in perimenopausal depression. The second aim is to examine whether reproductive hormones have an impact on the levels of acute-phase response proteins in perimenopausal depression. Lastly, possible effects of SSRIs on acute-phase response proteins levels are examined.

## Methods

### Subjects

A total of 65 women that were recruited consecutively as a case series from the Outpatient Clinics and the Departments of Obstetrics, Gynaecology and Psychiatry of the University Hospital of Larissa, Greece, participated in the study. Normal controls included women, who visited the outpatient department of Gynaecology, in order to take a Pap test, as a routine examination. All participants were Caucasian and they were divided into two groups. The first group consisted of perimenopausal women suffering from depression (n = 39) and the second one involved perimenopausal women without depression (n = 26). All women were in the perimenopausal phase, characterized by the presence of irregular cycles or amenorrhea for less than 12 months. The serum levels of follicle stimulating hormone (FSH) were over 20 IU/l for each participant in the study [37].

Furthermore, the exclusion criteria were the following: the presence of bipolar disorder, other psychiatric diseases (e.g. schizophrenia), diseases that can affect the immune system or cause depression (e.g. rheumatoid arthritis), the use of medications, except for SSRIs, recent stressful situations, such as bereavement, hysterectomy, oral contraceptives and hormone replacement therapy.

19 out of 39 women with depression had a history of depression and they were taking SSRIs, such as citalopram, fluoxetine or sertraline alone. Each woman had been taking SSRIs for more than a month without any other psychotropic or non-psychotropic medication. These women had experienced more than two episodes of depression in the past and they were still depressed, when enrolled into the study (17-item Hamilton Depression Rating Scale (HAM-D 17) score >10). Consequently, this subgroup of depressed perimenopausal women treated with SSRIs was classified as patients resistant to this class of antidepressants and therefore still depressed. The remaining 17 women had their first episode of depression during perimenopause and they had never used psychotropic medications. Depression was diagnosed through a psychiatric interview and the administration of the HAM-D 17. A woman would have been considered depressed, if she had scored over 10 on the HAM-D 17 and had fulfilled the criteria of major depression according to *International Classification of Diseases*, tenth edition (ICD-10). If a woman had scored less than or equal to 10 on the HAM-D 17, she would have been considered normal.

Venous blood samples were collected from 8.00 a.m. until 12.00 a.m. Samples were centrifuged at 3,500 rpm for 5 minutes and aliquots of serum samples were stored at -80°C until further use.

Informed consent for participation in this study was obtained from each woman. The ethics committee of the University Hospital of Larissa approved this study.

### Measurement of inflammatory markers

CRP, HP, Tf, C3, C4 and AAT concentrations were determined by laser nephelometry (Nefelometer, BN II System, Dade Behring). All antisera, controls and standards used for protein measurement were also obtained from Dade Behring. We used a single batch of antiserum for each parameter and included appropriate controls in each run.

### Statistics

Data analysis was conducted using the commercially available computer software SPSS V.15.0 (SPSS Inc., Chicago, IL, USA). The normality assumption was checked using the Shapiro test. When departures from normality were significant, non-parametric methods were used. In this case, the differences in acute-phase response protein concentrations between depressed and non-depressed perimenopausal women were analysed with the Mann–Whitney *U* test. On the contrary, when the data followed the normal distribution, *t*-test was used. Associations between continuous variables were tested using Pearson’s or Spearman’s correlations. Linear regression analyses were performed in order to find out if age, profession, education, marriage, months of amenorrhoea, smoking and alcohol intake affected the levels of acute-phase response proteins. A difference between two groups was considered to be statistically significant, when p < 0.05.

## Results

The major characteristics of perimenopausal women, with or without depression, are summarized in Table 1. The two groups did not differ in terms of age, years of education, months of amenorrhea, smoking habits, alcohol intake or marital status. As was expected, depressed women scored higher on the HAM-D 17 than women without depression (Table 1). Linear regression analyses showed that the levels of CRP, AAT, HP and C4 were not affected by age, professional, education, marriage, months of amenorrhoea, smoking and alcohol intake. On the contrary, it was found that the levels of TRf were affected by the profession (t = 2.51, p = 0.01) and marriage (t = 2.17, p = 0.03), C3 levels were influenced by the age (t = 2.37, p = 0.02) and smoking (t = 2.12, p = 0.04).

**Table 1:** Demographic characteristics of women with or without perimenopausal depression

	**Perimenopausal women with depression (n = 39)**	**Perimenopausal women without depression (n = 26)**	***p***
Age	50.10 ± 3.95	48.29 ± 10.95	0.94 (ns)^a^
Education (years)	8.22 ± 3.87	8.29 ± 3.93	0.94 (ns)^a^
Amenorrhea (months)	6.88 ± 4.62	7.10 ± 3.94	0.59 (ns)^a^
Married	36 (87.8%)	22 (91.7%)	0.37 (ns)^b^
Current smokers	15 (36.6%)	7 (29.2%)	0.6 (ns)^b^
Alcohol users	1 (0.02%)	0 (0%)	1 (ns)^b^
HAM-D	16.85 ± 5.02	5.68 ± 2.52	<0.0001^a^

^a^Mann–Whitney; ^b^Chi-Square test; ns, not significant; HAM-D, Hamilton Depression Rating Scale.

### Association between acute-phase response proteins and perimenopausal depression

The results of TRf (*t*-test, t = -0.534, df = 63, p = 0.595) AAT (*t*-test, t = -1.531, df = 63, p = 0.131) HP (*t*-test, t = 0.105, df = 63, p = 0.917) and C3 (*t*-test, t = 0.031, df = 63, p = 0.976) for individual cases of perimenopausal women, with or without depression, are shown in Table 2, whereas the results for C4 (Mann–Whitney U, Z = -0.763, p = 0.445) and CRP (Mann–Whitney U, Z = -0.521, p = 0.603) are depicted in Table 3. The concentration of acute-phase response proteins of women with depression did not differ significantly from those of normal controls.

**Table 2:** Serum TRf, AAT, C3 and HP concentrations in 39 women with perimenopausal depression, compared to those women without perimenopausal depression (n = 26)

	**Perimenopausal women with depression**	**Perimenopausal women without depression**	***P*-value**
	**(n = 39)**	**(n = 26)**	***t*-test**
TRf (mg/dl)	259.97 (47.54)	253.65 (45.52)	0,595 **ns**
AAT (mg/dl)	153.95 (23.93)	144.73 (23.55)	0,131 **ns**
C3 (mg/dl)	147.41 (44.62)	147.73 (33.21)	0,976 **ns**
HP (mg/dl)	142.66 (45.58)	143.91 (49.02)	0,917 **ns**

All results are expressed as mean (SD); ns, not significant.

**Table 3:** Serum C4 and CRP concentrations in 39 women with perimenopausal depression, compared to those women without perimenopausal depression (n = 26)

	**Perimenopausal women with depression**	**Perimenopausal women without depression**	***P*-value**
	**(n = 39)**	**(n = 26)**	**Mann-Whitney**
C4 (mg/dl)	32.80 (18.60-48.90)	29.20 (19.06-47.14)	0.445 **ns**
CRP (mg/dl)	0.20 (0.00-0.60)	0.10 (0.00-0.99)	0.603 **ns**

The results of C4 and CRP are expressed as medians (10-90%); ns, not significant.

### The effect of SSRIs on acute-phase response protein concentration

It was examined whether the depressed women, who were taking SSRIs, had different levels of acute-phase response proteins compared to depressed women, who were not taking SSRIs, and to women of normal controls. The results demonstrated that the levels of TRf (ANOVA F = 0.718, p = 0.492), AAT (ANOVA F = 2.365, p = 0.102), HP (ANOVA F = 0.085, p = 0.918) and C3 (ANOVA F = 1.685, p = 0.194) did not differ between the three groups (one-way ANOVA, Table 4). The same result was found for C4 (Kruskal-Wallis chi-square = 3.119, df = 2, p = 0.210) and CRP (Kruskal-Wallis, chi-square = 0.624, df = 2, p = 0.732) (Kruskal-Wallis, Table 5).

**Table 4:** Serum TRf, AAT, C3 and HP concentrations in 39 women with perimenopausal depression, subdivided to those receiving (n = 17) or not (n = 22) selective serotonin reuptake inhibitors (SSRIs) compared to those women without perimenopausal depression (n = 26)

	**Perimenopausal with**	**Women depression**	**Perimenopausal women without depression**	***P-*** **value (ANOVA)**
	**Not treated with SSRIs**	**Treated with SSRIs**		
	**N = 22**	**N = 17**	**N = 26**	
TRf (mg/dl)	248.59 (43.35)	267.65 (45.38)	253.65 (45.52)	0.492 **ns**
AAT (mg/dl)	149.14 (24.03)	158.71 (25.33)	144.73 (23.55)	0.102 **ns**
C3 (mg/dl)	138.19 (50.10)	158.24 (35.39)	147.73 (33.21)	0.194 **ns**
HP (mg/dl)	145.14 (46.67)	142.12 (39.91)	143.91 (49.02)	0.918 **ns**

All results are expressed as mean (SD); ns, not significant.

**Table 5:** Serum C4 and CRP concentrations in 39 women with perimenopausal depression, subdivided to those receiving (n = 17) or not (n = 22) selective serotonin reuptake inhibitors (SSRIs) compared to those women without perimenopausal depression (n = 26)

	**Perimenopausal with**	**Women depression**	**Perimenopausal women without depression**	***P-*** **value (Kruskal-Wallis)**
	**Not treated with SSRIs**	**Treated with SSRIs**		
	**N = 22**	**N = 17**	**N = 26**	
C4 (mg/dl)	37.68 (17.97-47.86)	33.75 (23.26-47.86)	29.20 (19.06-47.14)	0.210 **ns**
CRP (mg/dl)	0.23 (0.00-0.64)	0.26 (0.00-0.76)	0.10 (0.00-0.99)	0.732 **ns**

The results of C4 and CRP are expressed as medians (10-90%); ns, not significant.

### Hormone levels and cycle irregularity in relation to the presence of a first- ever depressive episode in perimenopause

It was studied whether women, who had their first-ever episode of depression in perimenopause differed as to their hormonal levels and months of amenorrhoea from depressed perimenopausal women, who had a history of depression, and from women of normal controls. The results showed that there were no differences in the levels of FSH (one-way ANOVA, F = 2.175, p = 0.12), of E_2_ (Kruskal-Wallis, *χ*^2^ = 0.288, p = 0.866) and months of amenorrhoea (Kruskal-Wallis, *χ*^2^ = 2.038, p = 0.361) between the three groups. On the contrary, depressed women with a history of depression had lower levels of LH (one-way ANOVA, F = 5.272, p = 0.007) (Table 6).

**Table 6:** **Serum FSH, LH, E**_**2**_**and months of amenorrhoea in women with the first episode of depression in perimenopause, in women with history of depression and in normal controls**

	**Controls**	**History of depression**	**First episode depression**	**p**
FSH	74.90 (30.11)^a^	63.16 (29.63)^a^	84.02 (30.66)^a^	0.12 (ANOVA)
LH	36.89 (18.49)^a^	27 (11.23)^a^	37.20 (11)^a^	**0.007 (ANOVA)**
E2	10.15 (19.2-37.84)^b^	8.47 (3.7-61.71)^b^	9.54 (2.33-49.77)^b^	0.866 (K-W)
Months of amenorrhoea	7 (3–12)^b^	4 (1–12)^b^	11 (1–12)^b^	0.361 (K-W)

^a^Mean(SD).

^b^Median (10-90%).

K-W: Kruskal-Wallis. Bold means statistical significance.

### Relationship between reproductive hormones and acute-phase response proteins

**Figure 1:**
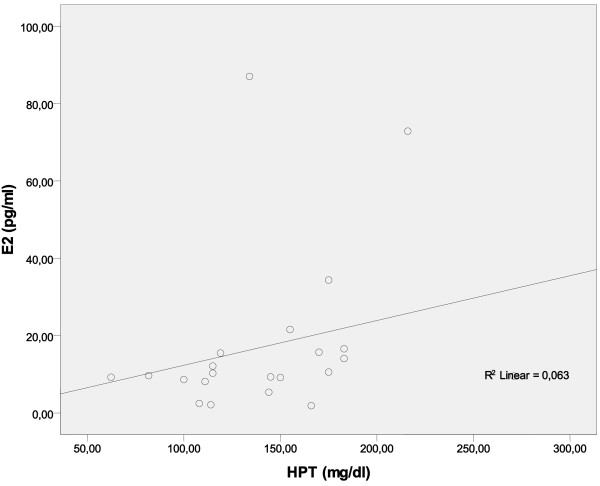
There is a positive correlation between haptoglobin (HPT) and E2 in perimenopausal women with depression who were not taking SSRIs.

It was investigated whether reproductive hormones affected the levels of acute-phase response proteins. The results showed that there was not any statistically significant correlation (Spearman’s correlation) between CRP, TRf, HPT, C3, C4, AAT and FSH, LH and E2 in the whole population (Table 6). This lack of a statistically significant correlation was also observed in depressed perimenopausal women, who were taking SSRIs (Table 7). On the contrary, there was a positive correlation between HPT and E2 (r = 0.480, p = 0.024, Table 8, Figure 1) in depressed perimenopausal women, who were not taking SSRIs.

**Table 7:** Relationship between CRP, TRf, HP, C3, C4, AAT and FSH, LH and E2 in the whole population (Spearman’s correlations)

	**C3**	**HP**	**C4**	**CRP**	**AAT**
FSH	r = -0.116	r = 0.056	r = -0.048	r = 0.045	r = 0.080
	p = 0.358	p = 0.658	p = 0.701	p = 0.721	p = 0.524
LH	r = -0.018	r = -0.066	r = -0.027	r = 0.025	r = -0.040
	p = 0.886	p = 0.602	p = 0.829	p = 0.843	p = 0.749
E2	r = 0.071	r = -0.064	r = 0.015	r = -0.164	r = -0.179
	p = 0.577	p = 0.615	p = 0.903	p = 0.196	p = 0.158

**Table 8:** Relationship between CRP, TRf, HP, C3, C4, AAT and FSH, LH and E2 in depressed perimenopausal women who were taking SSRIs (Spearman’s correlations)

	**C3**	**HP**	**C4**	**CRP**	**AAT**
FSH	r = -0.057	r = 0.285	r = 0.283	r = -0.213	r = 0.057
	p = 0.823	p = 0.251	p = 0.255	p = 0.397	p = 0.823
LH	r = -0.049	r = 0.258	r = 0.112	r = 0.198	r = 0.003
	p = 0.848	p = 0.301	p = 0.660	p = 0.431	p = 0.990
E2	r = 0.015	r = -0.310	r = 0.060	r = 0.236	r = -0.178
	p = 0.951	p = 0.211	p = 0.813	p = 0.346	p = 0.480

## Discussion

The main findings of this study in Caucasian women is that the positive acute-phase response proteins AAT, HP, CRP, C3 and C4 are not significantly increased and the negative acute-phase response protein TRf is not notably decreased in perimenopausal depression. Moreover, there is a positive correlation between HP and E2 in perimenopausal women with depression, who are not taking SSRIs. This finding does not add strength to the inflammatory theory of depression, which supports the view that positive acute-phase response proteins are increased and negative acute-phase response proteins are decreased in major depression.

The findings that stressors may be able to activate the release of pro-inflammatory cytokines and an acute-phase response in the absence of an immune challenge further support the notion that the immune system may be recruited to participate in the behavioural response to stress and therefore may contribute to the biochemical and molecular biological changes that characterize depression. It is thought that the acute-phase response in patients with major depression is related to increased production of pro-inflammatory cytokines, such as IL-1 and IL-6. IL-1 and IL-6 are pleiotropic cytokines, which are known to be major modulators of the acute-phase response and which may increase the synthesis of positive acute-phase proteins, while decreasing that of negative acute-phase proteins [20]. Nevertheless, in a previous study of ours with the same sample of the present study, it was found that in perimenopausal depression the concentration of pro-inflammatory cytokines is not increased [38]. This is in accordance with the present study, which shows that positive acute-phase response proteins do not also increase in this type of depression. However, the study of Ushiroyama et al. [39] measured IL-6 in a large sample of non-Caucasian patients and found increased plasma levels of IL-6 in the subgroup of women with depression and hot flashes, when compared to women with hot flashes without depression and to control subjects.

With regard to the reproductive hormone levels, it was found that women with a history of depression had lower levels of LH in comparison to women, who had their first-ever depressive episode in perimenopause. This finding is in accordance with a previous study, which supported that women with first-onset depressive episode during perimenopause had increased levels of LH [32].

Our study also showed a positive correlation between HP and E2 in depressed women, who were not taking SSRIs. There is a lot of conversation about the effects of estrogens on inflammation, neurodegeneration and mental health of menopausal women. It was believed that the use of estrogens contributed to the protection of neurons against degeneration, until the publication of the results of Women’s Health Initiative Memory Study (WHIMS) [40,41]. WHIMS indicated that women who received hormone therapy had a two-fold greater risk of developing Alzheimer’s disease than women in the placebo arm of the randomised double-blind clinical trial. Analyses of the estrogen-only therapy arm of the WHIMS trial indicated that women, who received conjugated equine estrogens, were not statistically different from women in the placebo arm of the trial, but there was a trend towards greater risk of Alzheimer’s disease and mild cognitive impairment.

Brinton [42] gave an explanation for these contradictory effects of estrogens on neurodegeneration. He claimed that the effects of estrogens depend on the health status of neurons. In the WHIMS cohort of women aged 65 and over with no indicators of neurological disease but with variable health status, who have been taking estrogen and hormone therapy for 5 years, presented an increase in the risk of developing Alzheimer’s disease. These data would suggest that as the continuum of neurological health progresses from healthy to unhealthy, the benefits of estrogen therapy are reversed and the estrogen therapy leads to the neural cell damage. If neurons are healthy at the time of estrogen exposure, their response to estrogen is beneficial for both neurological function and survival. On the contrary, if neurological health is compromised, estrogen exposure over time exacerbates neurological demise.

This theory seems to give an explanation for our result of the positive correlation between HP and E2 in depressed perimenopausal women, who were not taking SSRIs. Depression causes neurodegeneration and is associated with cognitive decline and memory problems, which in severe cases of major depression take the form of pseudodementia. This neurodegeneration of depression is mediated through inflammation. According to Brinton, in case of depression, when neurological health is compromised, estrogen exacerbates neurological demise.

Notwithstanding, the positive association between HP and E2 was not present in depressed women who were taking SSRIs. An explanation for this might be the fact that SSRIs have neuroprotective effects and therefore the neurons of these women are not so degenerated like the neurons of depressed women, who were not treated with SSRIs. One of the most widely used SSRI is fluoxetine. Several lines of evidence have shown that fluoxetine possesses potent neuroprotection against hypoxic-ischemia brain injury in rat pups [43], 3-4-methylenedioxymethamphetamine-induced neurotoxicity of the serotonin transporter in rat brains [44] and kainic acid-induced neuronal death in the mouse hippocampus [45]. Moreover, fluoxetine has been found to modulate neural stem cell survival and serotoninergic differentiation through modulating Bcl-2 expression [46] as well as to protect neurons against microglial activation and subsequent release of multiple pro-inflammatory and cytotoxic factors [47]. Recent studies have indicated that fluoxetine affords robust neuroprotection in the post-ischemic brain through its anti-inflammatory effect [48]. Furthermore, the long-term effect of antidepressants on the adult brain has been reported to be associated with increased neurogenesis, dendritic arborisation and synaptogenesis [49]. These actions of SSRIs are mediated through the MAP-kinase signal transduction pathway (by phosphorylation of ERK) and by stimulating neurotrophic factors, such as the brain-derived neurotrophic factor (BDNF) and the neuroprotective protein Bcl-2 [50].

Erdem et al. found that melancholic major depressed patients’ serum Hp concentrations were higher than non-melancholic major depressed patients and healthy controls. They also mentioned that there was a positive correlation between depression severity and serum Hp concentrations among the major depressed patients [51]. Therefore, the type and severity of depression affect the serum concentration of acute-phase response proteins. This finding is in conformity with our results, in a manner that in our study the depressive symptoms of perimenopausal depressed patients were not so severe (mean HAM-D = 16.8). Generally, perimenopausal depression is a type of a moderate depression as to severity, because depressive symptoms do not fulfil the complete clinical picture of major depression, either because there are not sufficient symptoms or they are not severe enough [52].

The present study has several limitations. First of all, perimenopausal depression has not been identified as a clinical entity and it is not included in the DSM-IV-TR. This study is a preliminary report and its data should be confirmed by studies that include larger samples of patients and controls. In addition, the diagnosis of depression was not based on a semi-structured interview but on a clinical interview and use of HAM-D 17.

## Conclusions

In conclusion, the lack of association of acute-phase proteins and depressive mood mentioned in this study does not support previous findings in patients with major depression. This negative finding in perimenopausal depression indicates either the absence or a more complex nature of the interactions between acute-phase proteins, low-grade inflammation and depression. The hormonal profile of women is a part of this complexity, because it seems that in perimenopause the hormonal changes are accompanied by changes of acute-phase response proteins. Particularly, in perimenopausal depression, there is an interaction between HP and E2, only in women who were not treated with SSRIs. The neuroprotective role of these drugs may protect depressed women from the activation of inflammation by increased levels of estrogens. Therefore, it seems that perimenopause is a period of a woman’s life during which hormonal, immune and metabolic changes occur and interact with each other making women vulnerable to depression.

## Abbreviations

AAT: a_1_-antitrypsin; APP: Acute-phase proteins; A2M: α_2_-macroglobulin; CRP: C-reactive protein; C3: Complement protein 3; C4: Complement protein 4; E2: Estradiol; FSH: Follicle stimulating hormone; HAM-D: Hamilton depression rating scale; HP: Haptoglobin; HRT: Hormone replacement therapy; IL-1: Interleukin-1; IL-6: Interleukin 6; LH: Luteinizing hormone; MD: Major depression; OB: Olfactory bulbectomized; SSRIs: Selective serotonin reuptake inhibitors; TRf: Transferrine; LH: Luteinizing hormone; E2: Estradiol.

## Competing interests

The authors declare that they have no competing interests.

## Authors’ contributions

SEK: design, interpretation, preparation of manuscript, editing, revising KAR: laboratory work AAR: recruitment, preparation of manuscript, revising NVA: conception of the study, overall coordination. All authors read and approved the final manuscript.

## Pre-publication history

The pre-publication history for this paper can be accessed here:

http://www.biomedcentral.com/1471-244X/14/164/prepub
